# Editorial: Dynamic systems theory and embodiment in psychotherapy research. A new look at process and outcome

**DOI:** 10.3389/fpsyg.2015.00914

**Published:** 2015-07-01

**Authors:** Sergio Salvatore, Wolfgang Tschacher, Omar C. G. Gelo, Sabine C. Koch

**Affiliations:** ^1^Department of History, Society and Human Studies, University of SalentoLecce, Italy; ^2^University Hospital of Psychiatry and Psychotherapy, University of BernBern, Switzerland; ^3^Department of Psychotherapy Science, Sigmund Freud UniversityVienna, Austria; ^4^Department of Therapy Sciences, SRH University HeidelbergHeidelberg, Germany

**Keywords:** dynamic systems theory, embodiment, psychotherapy process, psychotherapy outcome, self-organization, emergence, enactivism, temporality

This research topic encompasses a collection of articles from a dynamic systems and embodiment perspective on psychotherapy research. The collection follows the general tenet that communicative processes in psychotherapy are a field-dynamic phenomenon with temporal extension occurring in a context.

The context of psychotherapy, at any point in time, is given by multiple elements that belong to different phenomenological domains (e.g., sensation, behavior, affectivity, thought, language) and interact with each other and the environment over time (Salvatore and Tschacher, 2012). What works is the interaction between elements—namely, their being part of a whole—rather than the elements themselves. Consequently, no element is considered to possess invariant clinical meaning; rather, its impact on the entire therapeutic system is mediated by the field, understood as the set of ever-changing, co-occurring elements regulating (e.g., “enslaving”) the system's behavior. In addition, psychotherapy unfolds irreversibly through time. Everything happening within the communication between client and therapist (and within their minds) occurs in a time-frame, i.e., owed to what happened before, and paving the way for what will follow. In this sense, psychotherapy—just as any form of interaction—is inherently dynamic, and as such time-dependent.

Although these observations are familiar to clinicians, they have been widely neglected by researchers who have continued to endorse reductionist approaches (e.g., Elliott and Anderson, 1994). This is partly so because alternative approaches entail epistemological and methodological difficulties. Viewing psychotherapy in terms of field dynamics raises the epistemological issue of downward causality, i.e., the problem of modeling the *pars-toto* relation among levels of explanation. Moreover, the time dependency of psychotherapy processes renders most traditional strategies of data analysis unsuitable because these strategies commonly assume independent observations.

*Dynamic Systems Theory* (DST) (Thelen and Smith, 1994; Kelso, 1995; Haken, 2010) can offer a solution to this impasse. DST has developed in various fields (e.g., physics, biology, as well as cognitive sciences), adopting a holistic and time-dependent approach. However, it is not widely applied in psychotherapy research. The reason may be sought in the fact that DST represents a challenge for the traditional, evidence-based approach to the empirical study of psychotherapy. Psychotherapy research adopts mainly an inductive, data-driven logic of investigation. Accordingly, research is assumed to deal with facts, with interpretation following after. DST challenges such a view. It proposes a new way of looking at the relation between theory and data: Data are not self-contained facts ready to be retrieved and evident in and of themselves. Rather, they are the product of the theory-driven modeling of phenomena. The very notion of time dependency shows the inherent nexus between theory and data characterizing the DST perspective. Indeed time dependency is not an empirical fact, but a theoretical tenet that is used to interpret phenomena; accordingly, what is relevant is not the event that occurs, but the co-occurrence of the event with what occurs *before*, *together with*, and *after* it. Thus, it is a theoretical tenet that defines what empirical content to focus on: the co-occurrence of events. In the final analysis, the empirical datum of co-occurrence emerges only through and within the theoretical framework of the time-dependency tenet.

The theory-driven logic contained in DST provides a two-fold opportunity. On the one hand, it demands *methodological* innovation in the field of psychotherapy research. Data can be collected by making use of traditional instruments (e.g., session reports, category systems, video analysis, repeated ambulatory assessments, etc.) with a data analysis focus on measures of variability (e.g., standard deviation, entropy), since this is considered informative of the behavior of a dynamic system. Moreover, research designs should necessarily be longitudinal, aiming at assessing many time-points as possible over sessions and/or treatments. Finally, data-analysis should make use of longitudinal modeling in order to model the time-dependent system's behavior; moreover, idiographic approaches should be adopted, with the aim of being able to create general, nomothetic models without disregarding the individual, idiographic nature of each system's dynamics (e.g., Tschacher and Ramseyer, 2009).

On the other hand, it pushes researchers to develop *theoretical* frameworks capable of grounding the empirical investigation of clinical phenomena. The need for theoretical development is particularly evident in process research. Indeed, basic questions of outcome research (e.g., Does the psychotherapy work? For whom? Under which conditions?) may be addressed in terms of the evidence-based paradigm, this does not hold once the focus moves to the issue of *why* and *how* psychotherapy works. Answers to such questions require developing a model of psychotherapy process—an enterprise that cannot be carried out purely empirical, i.e., as a mere accumulation of evidence. Theory-free research has provided an increasing collection of factors that play a role (moderating, mediating) in clinical exchange and its efficacy; and this has been enlarging the knowledge of what is relevant in psychotherapy process. However, this process in itself—the inherent dynamics of how it works—has remained a black box. The more data one collects, the more one is able to detect what happens outside the box—the input, the output, and their linkage—but one cannot look inside. The key to open the black box is theory, not data.

In this situation, some clinical researchers have started to introduce ideas of *embodiment and enaction* into psychotherapy research (e.g., Fuchs and Schlimme, 2009; Koch, 2011; Michalak et al., 2014). Embodiment and enaction theories bring in an organismic perspective on human interaction and outcomes—taking into account body-environment coupling, dynamic movement, emergent phenomena, and the circularity of interaction processes as opposed to the cognitivistic computer metaphor that tries to predict interaction processes and outcomes from a linear causality perspective. This innovative view triggers a rethinking of the clinical interaction by recognizing the embodied nature of psychological and communicative phenomena. The embodied enactive perspective has extended the cognitive paradigm in psychology to include the body, that is, the “lived body” as conceptualized by phenomenology (e.g., Merleau-Ponty, 1962), as an organismic, self-organizing entity (Varela et al., 1992), forming multiple feedback cycles with its environment (Gibson, 1966). Empathy, bonding and rapport are formed on a body basis (Ramseyer and Tschacher, 2011).

The tenet of embodiment contributes to the theoretical framework psychotherapy research has been looking for. The integration of this tenet with DST opens up a promising scenario in the field of psychotherapy research, developing new transdisciplinary theoretical concepts, methodologies, and standards of knowledge. The notion of field dynamics enables us to account for the role played by the communication context in the regulation of intra-psychological processes. Moreover, the new embodied-systemic approach provides a way of seeing psychological phenomena in terms of dynamic Gestalts, thereby enabling researchers to go beyond hampering dichotomies (e.g., mind-body; structure-function) as well as beyond reductive, molecular approaches. The embodied-systemic approach is prone to develop methodological strategies transcending the conventional opposition between idiographic and nomothetic sciences, by accounting for the temporal dynamics of data.

This research topic aims to outline and develop this promising scenario. We have collected theoretical, methodological, and empirical papers that highlight the heuristic power of approaches endorsing the embodied and field-dynamic nature of clinical phenomena. In sum, these contributions demonstrate the need for (a) more theory development in the field of psychotherapy research, (b) more development of methods that appropriately reflect the complexity of natural interaction between two or more agents, and (c) more translational research based upon clinical questions and implicit knowledge of clinical practitioners. We hope that this special issue is a beginning of clinicians and researchers being bolder in terms of acknowledging complexity, emergence and uncertainty, developing theories, methods and practice that account for them. The collected contributions pave the way for more appropriate and heuristically more powerful empirical investigations of complex phenomena such as the psychotherapy process.

## Conflict of interest statement

The authors declare that the research was conducted in the absence of any commercial or financial relationships that could be construed as a potential conflict of interest.
